# Associations between Periodontitis and COPD: An Artificial Intelligence-Based Analysis of NHANES III

**DOI:** 10.3390/jcm11237210

**Published:** 2022-12-04

**Authors:** Andreas Vollmer, Michael Vollmer, Gernot Lang, Anton Straub, Veronika Shavlokhova, Alexander Kübler, Sebastian Gubik, Roman Brands, Stefan Hartmann, Babak Saravi

**Affiliations:** 1 Department of Oral and Maxillofacial Plastic Surgery, University Hospital of Würzburg, 97070 Würzburg, Germany; 2 Department of Oral and Maxillofacial Surgery, Tuebingen University Hospital, Osianderstrasse 2-8, 72076 Tuebingen, Germany; 3 Department of Orthopedics and Trauma Surgery, Medical Centre-Albert-Ludwigs-University of Freiburg, Faculty of Medicine, Albert-Ludwigs-University of Freiburg, 79106 Freiburg, Germany; 4 Division of Medicine, Department of Oral and Maxillofacial Surgery, University Hospital Ruppin-Brandenburg, Brandenburg Medical School, Fehrbelliner Straße 38, 16816 Neuruppin, Germany

**Keywords:** COPD, periodontitis, bone loss, machine learning, prediction, artificial intelligence, model, gingivitis

## Abstract

A number of cross-sectional epidemiological studies suggest that poor oral health is associated with respiratory diseases. However, the number of cases within the studies was limited, and the studies had different measurement conditions. By analyzing data from the National Health and Nutrition Examination Survey III (NHANES III), this study aimed to investigate possible associations between chronic obstructive pulmonary disease (COPD) and periodontitis in the general population. COPD was diagnosed in cases where FEV (1)/FVC ratio was below 70% (non-COPD versus COPD; binary classification task). We used unsupervised learning utilizing k-means clustering to identify clusters in the data. COPD classes were predicted with logistic regression, a random forest classifier, a stochastic gradient descent (SGD) classifier, k-nearest neighbors, a decision tree classifier, Gaussian naive Bayes (GaussianNB), support vector machines (SVM), a custom-made convolutional neural network (CNN), a multilayer perceptron artificial neural network (MLP), and a radial basis function neural network (RBNN) in Python. We calculated the accuracy of the prediction and the area under the curve (AUC). The most important predictors were determined using feature importance analysis. Results: Overall, 15,868 participants and 19 feature variables were included. Based on k-means clustering, the data were separated into two clusters that identified two risk characteristic groups of patients. The algorithms reached AUCs between 0.608 (DTC) and 0.953% (CNN) for the classification of COPD classes. Feature importance analysis of deep learning algorithms indicated that age and mean attachment loss were the most important features in predicting COPD. Conclusions: Data analysis of a large population showed that machine learning and deep learning algorithms could predict COPD cases based on demographics and oral health feature variables. This study indicates that periodontitis might be an important predictor of COPD. Further prospective studies examining the association between periodontitis and COPD are warranted to validate the present results.

## 1. Introduction

The rise of chronic diseases is one of society’s most pressing challenges. The most significant risk factor is the lifestyle associated with stress, unhealthy eating habits, alcohol consumption, and tobacco use. A poor oral hygiene routine is related to periodontal disease as well as poor general health [1]. Inflammation of the gums and surrounding bone tissue causes periodontitis, which is a chronic condition that results in the loss of teeth. As a result of bacteria forming a biofilm as dental plaque, the immune system is stimulated, ultimately leading to irreversible loss of periodontal structures [2]. The formation of biofilms can directly influence the inflammatory process at the local level and indirectly through the stimulation of catabolic cytokines and inflammatory mediators in the body’s systemic circulation [2,3,4]. A growing body of literature focuses on the systemic effects of periodontitis and its consequences on general health [5]. Various chronic diseases, such as cardiovascular diseases, adverse pregnancy outcomes, diabetes, and rheumatic diseases, have been linked to periodontitis [6,7,8,9].

Chronic obstructive pulmonary disease (COPD) is another widespread disease that negatively impacts global health [10]. There are several similarities between COPD and periodontitis and the presence of chronic inflammation, which can have both systemic and local effects [11]. An altered microbiome in the lungs has been associated with an escalation of the production of inflammatory mediators, similar to the effects of periodontitis, which have a systemic as well as a local effect [12,13,14]. A systematic review has already found evidence that COPD is associated with poor oral health [15]. However, as a limitation, no subgroup analysis could be conducted since the study endpoints of the 14 included studies of varying quality were very different. Due to the differences in parameters examined, further studies will need to be conducted to confirm the correlation [15].

There appears to be a highly multifactorial connection between the two diseases. An investigation of immunomodulatory mechanisms was conducted in a recent systematic review [16]. It was noted by the authors that immune cells play an important role in both diseases. Neutrophils play an integral role in the imbalance of proteases and oxidative stress [16,17]. In addition, macrophages play a crucial role in the progression of the disease process on a systemic level. It appears that these cells have a similar impact on the severity of both disease entities. Recently, it was also demonstrated that dendritic cells play a major role in the development of both diseases [16,17]. It has also been suggested that genetic conditions associated with COPD, such as alpha-1 antitrypsin deficiency or rheumatoid arthritis deficiency (AATD), may cause predisposition to the disease. According to the authors, neutrophil inflammation can exacerbate the condition owing to an increased neutrophil function. It has been reported that this enhancement occurs in diseases such as periodontitis, COPD, and AATD [18,19]. A correlation between the aspiration of an anaerobic microorganism called Fusobacterium nucleatum, which is one of the key germs for the formation of a biofilm and the development of periodontitis, and the exacerbation of COPD was shown by Suzuki et al. [20]. The opposite effect with optimization of oral hygiene and reduction in the microbial load, and consequently a lower number of exacerbations, was shown in a systemic review from 2021 [21]. Generally, patients with high-risk constellations, such as hospitalized patients, are more at risk for bacterial colonization than outpatients [22,23]. Another mechanism is the production of inflammatory mediators such as IL-1α, IL-1β, IL-6, IL-8, and TNF-α, which can enter the systemic circulation and thereby maintain or trigger other chronic diseases [4,24,25]. A combination of the above-mentioned mechanisms can stimulate the production of further cytokines as well as the migration of inflammatory cells into the tissue through the production of cytokines located within the sulcus fluid on the one hand and aspirated saliva on the other [25,26,27,28]. As a consequence, an excess of enzymes is produced, which destroy the respiratory epithelium in a manner similar to what occurs in the gingiva, resulting in the progression of the disease [2,3,22,26,29]. There are other very interesting associations besides COPD, such as COVID-19, which primarily affects the respiratory system. Both periodontitis and COVID-19 have been associated with an exaggerated immune response. There is already evidence of an association between both disease entities in a case-control study, which highlights the importance of these associations in the scientific community [30]. Due to the multifactorial nature of these associations, molecular and genetic interactions are also an intriguing area of research.

Using artificial intelligence for large data analysis could be of great benefit due to its capability to identify patterns in large amounts of data that cannot be deciphered by basic statistical approaches [31,32]. A number of artificial intelligence-based algorithms have recently made significant advances due to the increasing digitalization of medicine and the emergence of databases with large amounts of data. A number of medical tasks are becoming increasingly accurate as a result of the ability to obtain accurate results from large training datasets and improvements in algorithms over time [33]. Nevertheless, learning requires well-documented databases containing a large number of data [34]. A wide variety of information is collected within the different clinics, and each clinic collects and documents information in a way that is often unique. Considering that individual institutes create databases with a specific aim, they are biased towards the institute creating the database. It is not possible to overcome this bias, even with modern artificial intelligence algorithms for noise reduction [35]. Although there has been a great deal of research, independent public databases are relatively uncommon. It would be beneficial to use a large open-access database of the general population in order to train an artificial intelligence based on a specific question.

The relationship between periodontitis and COPD has already been suggested by several studies [36,37,38]. In spite of this, they have not been able to provide a conclusive explanation to date, although multiple confounding variables were considered in multivariate analyses [39]. The present study uncovers hidden links between COPD and periodontitis in a large general population for the first time using artificial intelligence and biostatistical approaches.

## 2. Materials and Methods

### 2.1. Study Design

The Third National Health and Nutrition Examination Survey (NHANES III) surveyed the civilian population in the United States between 1988 and 1994. Multistage stratified sampling was used to select the sample population. The National Center for Health Statistics (NCHS) and the Center for Disease Control and Prevention (CDC) monitored and approved this survey. The data are available for research purposes and were extracted and processed for the present study.

According to NHANES III, this sample represents the total noninstitutionalized population in each of the 50 U.S. states aged 2 months and older. Generally, the NHANES III sample design follows the same structure as the previous National Health and Nutrition Examination Surveys. Stratified multistage probability designs were used in each of these surveys. As part of the design, an initial sample of 81 primary sampling units (PSUs), which are generally individual countries, was selected. To keep PSUs above a minimum size, adjacent counties were combined in some cases. A probability-proportional-to-size (PPS) method was used to stratify and select PSUs. In total, thirteen large counties (strata) were selected with certainty (probability of one). As a result of logistical and operational considerations, these 13 certainty PSUs were divided into 21 stands (survey locations). The remaining PSUs were divided into 34 strata after the 13 certainty strata were determined, and 2 noncertainty PSUs were chosen for each stratum. PPS was used in the selection process, without replacement. In addition, each noncertainty PSU was also referred to as a “stand;” therefore, NHANES III contained 81 PSUs or 89 stands. In addition to these steps, several other stages of sampling were conducted until the stage of “interviewing the sample person” and “examining the sample person”. The exact sampling process can be found in the report “Sample Design: Third National Health and Nutrition Examination Survey” [40].

**Figure 1:**
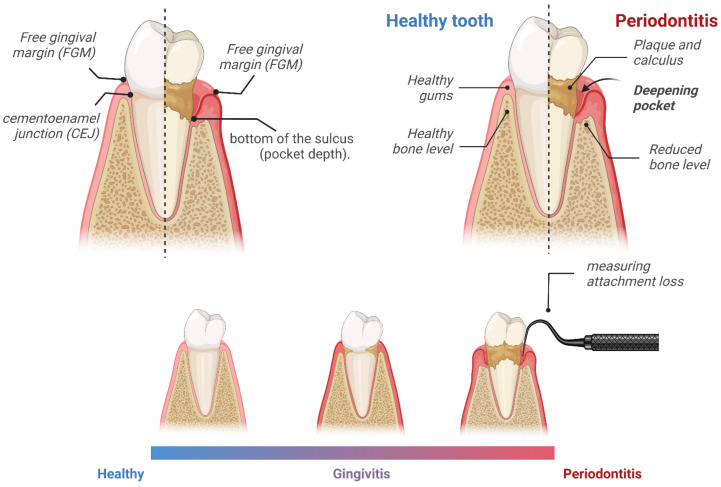
To investigate the extent of the attachment loss, measurements were taken with a probe. The first measurement was the distance between the free gingival margin (FGM) and the enamel–cement junction (CEJ) and the second was the difference between the FGM and the bottom of the sulcus (pocket depth) [41,42,43]. Illustration created with BioRender.com.

A licensed dentist who is specially trained in the use of specific epidemiologic indices of oral health conducted an oral and dental examination on subjects one year of age and older. In order to determine the degree of attachment loss (AL), two measurements were taken: (1) the distance between the free gingival margin (FGM) and the cementoenamel junction (CEJ), and (2) the distance between the FGM and the bottom of the sulcus (pocket depth). Those cases in which the gingival margin had receded and the CEJ was exposed indicated gingival recession. To calculate the attachment loss (level) variables, the distance between the base of the sulcus and the CEJ was subtracted from the distance between the FGM and the CEJ (Figure 1) [41,42].

Subjects eight years of age and older were subjected to pulmonary function tests (spirometry) by trained personnel at the mobile examination center. It was also possible to conduct the examination at home for subjects 60 years and older who were unable or unwilling to come to the mobile examination center. Prior to the examination, subjects were asked screening questions to determine whether they were excluded due to medical reasons. Exclusion from the study was made for individuals who had undergone chest or abdominal surgery within three weeks prior to the examination or had suffered from heart problems (myocardial infarction or heart attack, angina or chest pain, congestive heart failure) throughout the preceding six weeks.

### 2.2. Data Handling and Statistical Analysis

After extraction of the Excel table from the database (https://wwwn.cdc.gov/nchs/nhanes/nhanes3/default.aspx, accessed on 6 November 2022), the dataset was imported into the statistical program SPSS V. 18.0 (IBM Corp., Armonk, NY, USA). This study also included information on the subjects’ demographics and socioeconomic status (SES). Medical and demographic data were obtained by means of questionnaires and physical examinations by a physician. Age, gender, and ethnicity (categorized as white, black, Mexican-American, and other ethnicities) were the demographic variables included in this analysis. The poverty income ratio (PIR) was used as a measure of SES. Two components were used to calculate PIR. Using the Family Questionnaire, the numerator represented the midpoint of the observed family income category. Denominators included the poverty threshold, the ages of family reference individuals, and the year in which the family was interviewed. The lifestyle characteristics examined included smoking history (estimated by multiplying the number of years each subject smoked by the average number of packs smoked per day). American Thoracic Society recommendations were followed in performing the spirometry. Participants with forced expiratory volume in 1 s (FEV1)/forced vital capacity (FVC) <70% were diagnosed with chronic obstructive pulmonary disease.

In this analysis, “COPD diagnosis” (((FEV1)/(FVC) × 100) < 70%) was used as the dependent variable. Several independent variables reflecting oral health were considered for the investigation of COPD and periodontitis, including mean clinical attachment loss (MAL), gingival bleeding, number of furcations, and decayed/missing/filled teeth (DMFT)/decayed/missing/filled surfaces (DMFS). Descriptive statistics were performed on the data following the identification of all independent and dependent variables and covariates in order to examine possible associations between the general characteristics of the population. The Shapiro–Wilk test was performed on the continuous variables in order to determine whether they had a normal distribution. Pairwise analyses were employed to evaluate and compare the parameters studied between the COPD group and the non-COPD group. The proportion of subjects with and without COPD in relation to categorical independent variables was evaluated using contingency tables and chi-square tests.

The dataset was clustered based on the features in the dataset using unsupervised learning with k-means clustering. An automated clustering method was used based on Schwarz’s Bayesian criterion (BIC). Cluster variables were compared using the Mann–Whitney U test or chi2-test as appropriate. A multiple imputation approach was used to impute missing values. The target COPD classes were predicted using machine learning techniques and deep learning algorithms. In this study, logistic regression, random forest, stochastic gradient descent, k-nearest neighbors, decision tree, Gaussian naive Bayes (GaussianNB), support vector machines (SVMs), a custom convolutional neural network (CNN), a multilayer perceptron artificial neural network (MLP), and a radial basis function neural networks (RBNNs) were employed as classifiers. The Python code is available in the data availability section. The hardware and software environment specifications were as follows:

- CPU: AMD Ryzen 9 5950X 16-Core Processor (Santa Clara, CA, USA);
- RAM: 64 GB;
- GPU: NVIDIA Geforce RTX 3090 (Santa Clara, CA, USA);
- Python version: 3.10.4 (64-bit) (Wilmington, DE, USA);
- OS: Windows 10 (Redmond, WA, USA).

Statistical analyses were conducted in Python and SPSS v27 (IBM, Armonk, NY, USA).

## 3. Results

**Figure 2:**
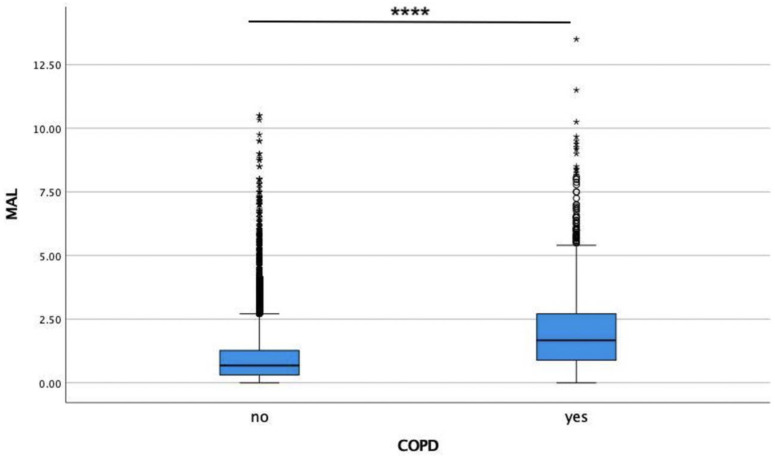
Comparison of the mean attachment loss (MAL) between the COPD and the non-COPD group. Asterisks (*) represent extreme outliers (3rd quartile + 3*interquartile range or 1st quartile − 3*interquartile range; circles represent moderate outliers (3rd quartile + 1.5*interquartile range or 1st quartile − 1.5*interquartile range). **** *p* < 0.0001.

An overall of 15,868 participants were included after data preprocessing. The mean age of participants was 40.42 ± 19.85 years (range: 13–90 years). Mean attachment loss significantly differed between the COPD (2.05 ± 1.64 mm) and the non-COPD (0.96 ± 1.03 mm) group (*p* < 0.0001) (Figure 2).

**Figure 3:**
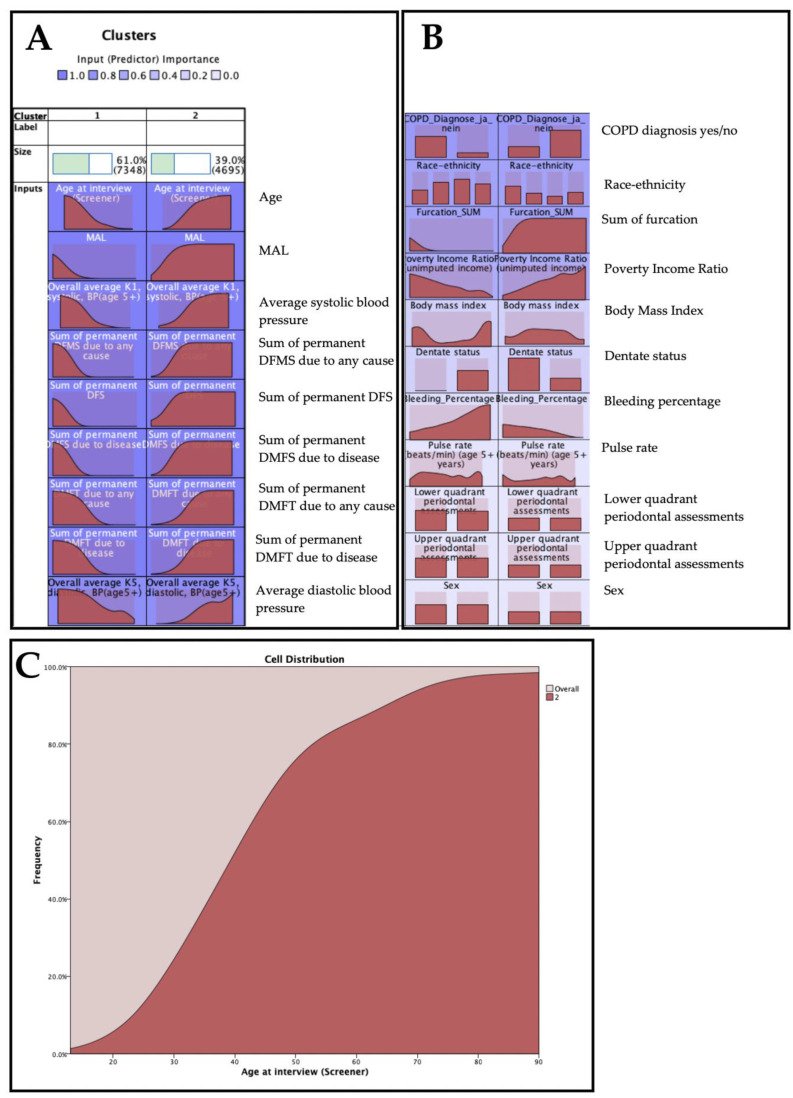
K-means cluster analysis, including the COPD class variable. The most important feature to classify the data was the age at interview, followed by the mean attachment loss (MAL), the sum of permanent DMFS due to disease (i.e., caries or periodontitis), and the sum of permanent DMFT due to disease. In the distribution charts, the distribution of the features is shown for both clusters. (**A**) illustration of most important predictors (feature importance ≥0.8; (**B**) illustration of less important predictors (feature importance <0.8); (**C**) In order to facilitate interpretation, an example feature is presented (age at interview). According to the selected feature, the age distribution is shifted to the right for the high-risk cluster while it is shifted to the left for the low-risk cluster.

To cluster the dataset into two classes, we performed unsupervised learning using k-means cluster analysis and evaluated which features are significant for clustering. There were two clusters as a result of the cluster analysis (39.0% in the high-risk group and 61.0% in the low-risk group) (Figure 3). There was a significant difference between the high-risk cluster and the low-risk cluster on several cluster variables (age, mean attachment loss (MAL), systolic blood pressure, the sum of permanent DMFT and DMFS due to disease, number of furcations, etc.) (*p* < 0.0001 for all comparisons). Notably, the high-risk group contained 85.0% of all COPD cases, whereas the low-risk cluster contained 15.0% of the COPD cases. Conversely, 34.5% of non-COPD instances were present in the high-risk group, whereas 65.5% were present in the low-risk group. In general, results revealed that there were two clusters depicting the patients’ risk levels (i.e., patients with a high-risk level had a higher age, a higher MAL, and a higher percentage of COPD cases).

Table 1 illustrates the feature distribution between high- and low-risk clusters. All participants who were edentulous in one arch were present in the high-risk group. Most patients having teeth were present in the low-risk group. All variables but sex (*p* = 0.814), upper quadrant periodontal assessment (*p* = 0.513), and lower quadrant periodontal assessment (*p* = 0.285) showed highly significant differences between the high-risk and low-risk clusters (*p* < 0.0001).

**Figure 4:**
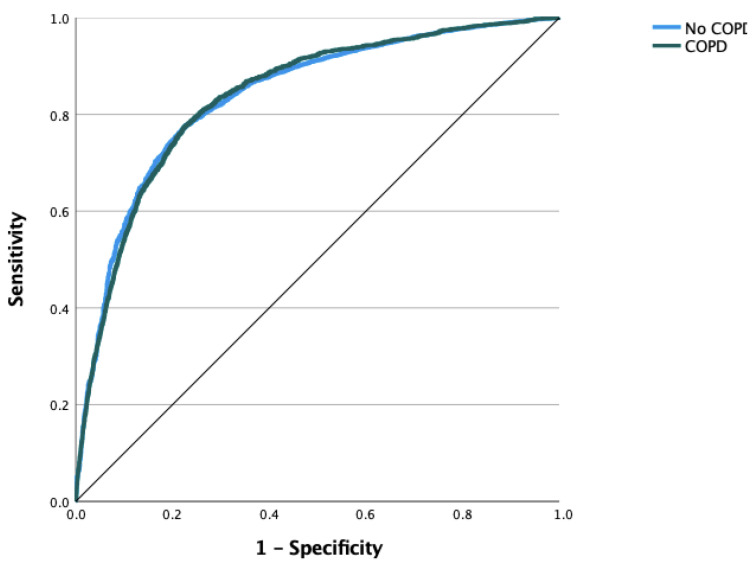
Prediction of COPD class with a multilayer perceptron (MLP) model. Input layer: feature variables (27 units). Hidden layer (5 units): activation function—hyperbolic tangent. Output layer: dependent variable—COPD, activation function—softmax, error function cross-entropy. The testing data criterion determined the number of units in the hidden layer: the best number of hidden units is the one that yields the smallest error in the testing dataset. AUC: 0.838 (fold 5 ROC curve).

**Figure 5:**
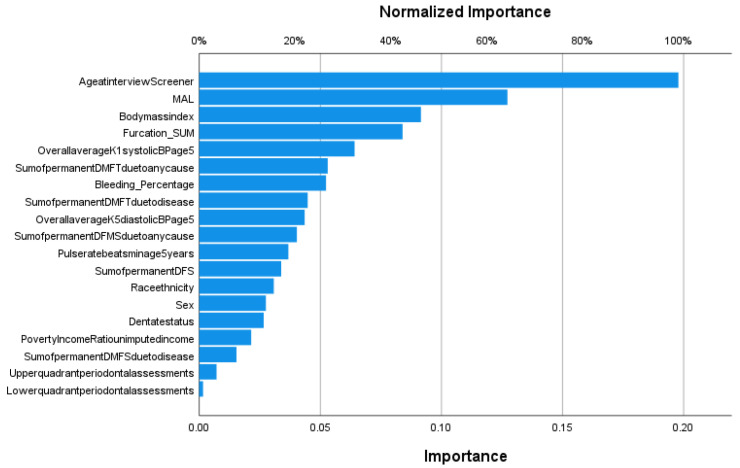
Feature importance analysis for predicting the COPD classes in the multilayer perceptron model. Variable names are shown as listed in the NHANES3 dataset.

The next step involved the application of machine learning and deep learning algorithms in order to predict COPD classes. Multilayer perceptrons achieved an AUC of 0.836 over fivefold cross-validation (Figure 4). Based on the feature importance analysis, age was found to be the most crucial factor for classification, followed by MAL, BMI, and the number of furcations (Figure 5).

The accuracies and AUC of the machine learning algorithms and the custom-made convolutional neural network (CNN) are shown in Table 2. The custom-made CNN reached the highest accuracy and AUC, whereas the decision tree classifier and the SVM performed worse with regard to the AUC.

**Figure 6:**
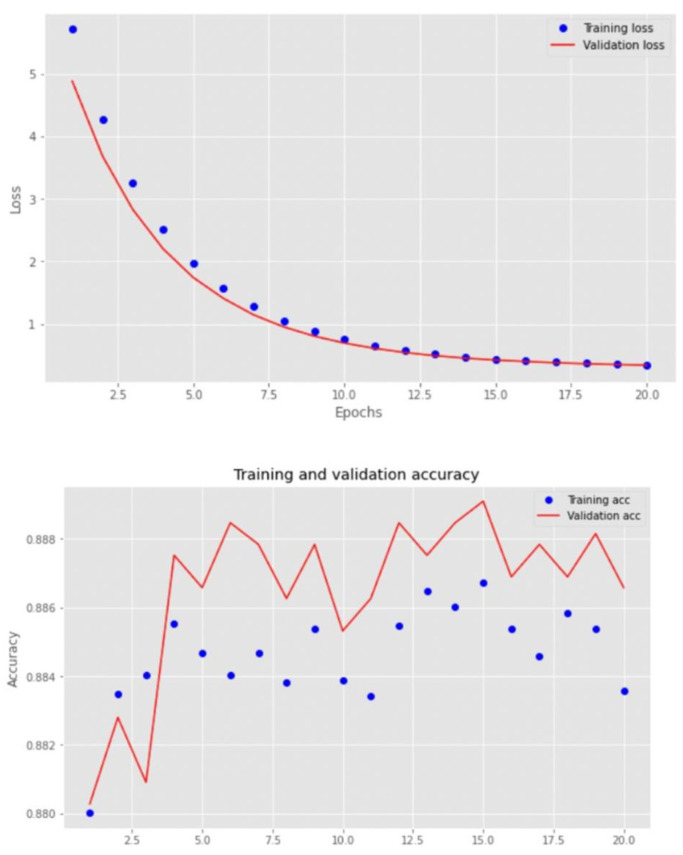
Loss and accuracy curves for the training and validation phase of the custom-made CNN model.

The loss curves during the training and validation phase for the best-performing algorithm (custom-made CNN) are shown in Figure 6.

## 4. Discussion

An extensive database containing standardized oral health and lung function evaluations was used for prediction modeling using machine learning and deep learning techniques. Based on sociodemographic factors and oral health variables, the algorithms presented in this study were capable of accurately predicting COPD classes. The results suggest that oral health parameters, including a high attachment loss, play a significant role in predicting the COPD class. Thus, based on the large dataset included in this study, COPD and periodontitis appear to be significantly related.

Despite the fact that a connection between periodontitis and COPD was also examined in the more recent NHANES data from 2009 to 2012 with non-AI-based methodologies, the analysis of the present work utilized older data from 1988 to 1994 [44]. As already mentioned, artificial intelligence requires a large amount of data in order to recognize the best possible analysis of hidden patterns [34]. Specifically, algorithms need a lot of different data to learn exactly what patterns they should or should not recognize [45]. Even though the data of the present study are older, they provide a larger amount of data with a total of 15,868 participants than the new data from 2009 to 2012 with a total of only 6313 subjects [44]. A comparison of the data of both studies would be desirable on the basis of further studies. It would also be interesting to generate a pooled data source from all available databases that would allow the maximum number of subjects for training an artificial intelligence. However, the different documentation within the various data sources must be taken into account here [46].

There is an increasing impact of chronic diseases on general health in today’s society [1,2]. It has been demonstrated in several studies that chronic inflammation plays a role in both periodontitis and chronic obstructive pulmonary disease (COPD) [6,7,8,9,11,12,13,14]. Several studies have already attempted to demonstrate a relationship between periodontitis and COPD or pneumonia [36,37,38,47,48]. In addition to this clear evidence, other authors have reported weak or no evidence of a relationship between periodontitis and COPD [49,50,51]. This discrepancy might be attributed to the different methodologies used in these studies. This discrepancy may also be explained by different diagnostic criteria for periodontitis and COPD [46]. This may also be due to the general limitations of the statistical approach used in most studies on this subject. A pairwise analysis, for example, has a high risk of overlooking the influence of other factors. Using an artificial intelligence approach for the analysis of the dataset has the advantage of detecting hidden patterns more effectively than using pairwise statistical analysis [46].

The present analysis may be affected by the possibility of polymedication among COPD patients. Steroids, for example, suppress inflammatory activity, which negatively impacts mucous membranes and periodontal appendages [46]. Polymedications were not considered in the NHANESIII dataset. As of yet, most studies carried out to date have used pairwise statical approaches to analyze their institute databases. Studies comparing COPD and non-COPD groups regarding periodontal health often did not include additional factors that might influence periodontal health, as demonstrated in a meta-analysis [39]. There are drawbacks to pooling small studies from different institutes in order to examine the relationship between COPD and periodontitis, such as different measurement techniques or limited comparability due to the different predefined study questions and the resulting different recruitment strategies [35]. By using artificial intelligence in this paper, we were able to uncover patterns that would have been hidden by simple pairwise analysis. The results of our study confirm the findings of previous meta-analyses on this topic, indicating that COPD and periodontitis are significantly related in the general population [16,39].

Due to the cross-sectional design of the NHANES III study, a longitudinal study of patients and an examination of the cause–effect relationship between COPD and periodontitis was not possible. Further, since the dataset was constructed using the US population, no generalizations can be made regarding the data for other races or populations [46]. As a result of the nature of the study, other relevant data, such as brushing behavior or frequency of dental visits, cannot be assessed, which limits the final interpretation [46]. However, such confounding variables may have an important impact on outcomes. In a randomized clinical trial, significant evidence of a lower incidence of COPD exacerbations was demonstrated for the group that received regular professional dental cleaning compared to the group that only received a dental examination [52]. Compared to basic statistical methods, such as pairwise analysis, sophisticated artificial intelligence-based algorithms are able to recognize many different variables with regards to their importance for a specific outcome [34,46]. However, the statistical approach chosen has some limitations as well. Predictions are based on the training dataset (US population), which limits their applicability to other populations. Additionally, there may be other suitable prediction algorithms that were not included in the present study. The chosen approach has the disadvantage of requiring a significant amount of resources for the learning and prediction process. The use of basic statistical methods in other software may be faster and more cost-effective in future studies that examine this relationship. Additionally, the chosen approach is highly dependent on the dataset. This may lead to different conclusions in future studies that include more variables. For example, data regarding alcohol and nicotine consumption were missing in many cases, limiting the analysis of their impact on COPD. In most cases, COPD is associated with tobacco use, and tobacco use is known to contribute to the progression of periodontal disease [37]. This study has several strengths, including a large number of subjects, providing a good overview of a particular cohort—in this case, the US population—and the numerous algorithms that were included for evaluation.

Our study demonstrated that poor oral health and age play a significant role in predicting COPD. Bacterial infection is a known risk factor for COPD, and exacerbations of COPD may be caused by bacterial infections [53,54]. The most common bacteria responsible for exacerbations are Haemophilus influenzae, Streptococcus pneumoniae, and Moraxella catarrhalis. A correlation was found between poor oral health and infections with these bacteria in patients with COPD by Scannapieco et al. [22]. An infection of the lungs may result from the inhalation of oral microorganisms through the saliva. This mechanism is responsible for the majority of anaerobic infections of the lung [22,55,56,57].

Studies also showed that if periodontitis is effectively treated, the measurable inflammation parameters can be reduced [4,52,58,59,60,61]. It is not possible to determine the direction of the relationship between periodontitis and COPD based on the results of the study. However, optimizing oral health has been shown to improve the disease activity of COPD [4,52] and our results support the theory that oral health improvements should be an important therapeutic pillar in COPD patients.

## 5. Conclusions

The findings of the study indicate that poor oral health is strongly associated with COPD. Based upon sophisticated artificial intelligence-based analyses, high CAL appears to increase the risk of COPD, although causal relationships cannot be concluded due to the cross-sectional design of the study. Providing a detailed understanding of the multivariate pathogenesis will require an analysis of all influencing factors, including other noxious agents, genetic predispositions, and concomitant diseases. The results of this study are of high interest to clinicians and epidemiologists considering the high prevalence of periodontitis and COPD. There is a need for large prospective longitudinal studies in order to examine the relationship in greater detail. Additionally, oral health should be considered an important therapeutic pillar in patients with COPD and those at risk of developing it.

## Figures and Tables

**Table 1:** Using the k-means cluster analysis, including the COPD class variable, descriptive statistics were calculated, and pairwise comparisons were made between the clusters.

		Low-Risk Cluster				High-Risk Cluster			
		Count	Row N %	Mean	Standard Deviation	Count	Row N %	Mean	Standard Deviation
COPD diagnosis	no	7187	65.5%			3780	34.5%		
	yes	161	15.0%			915	85.0%		
Race/ethnicity	Non-Hispanic white	1946	44.0%			2477	56.0%		
	Non-Hispanic black	2295	66.4%			1163	33.6%		
	Mexican-American	2786	76.3%			867	23.7%		
	Other	321	63.1%			188	36.9%		
Sex	Male	3525	61.1%			2242	38.9%		
	Female	3823	60.9%			2453	39.1%		
Upper-quadrant periodontal assessments	1	3694	60.7%			2389	39.3%		
	2	3654	61.3%			2306	38.7%		
Lower-quadrant periodontal assessments	3	3659	61.5%			2291	38.5%		
	4	3689	60.5%			2404	39.5%		
Dentate status	Completely edentulous	0	0.0%			0	0.0%		
	Edentulous in one arch	0	0.0%			177	100.0%		
	Teeth present	7348	61.9%			4518	38.1%		
Age at interview (Screener)				28	11			53	16
Poverty income ratio (unimputed income)				3	2			2	2
Pulse rate (beats/min) (age 5+ years)				74	12			75	12
Overall average K1, systolic, BP (age 5+)				114	12			129	19
Overall average K5, diastolic, BP (age 5+)				69	11			76	10
Body mass index				25.6	5.9			27.5	5.7
Sum of permanent DMFS due to disease				12	11			56	23
Sum of permanent DFMS due to any cause				13	11			58	23
Sum of permanent DFS				8	7			30	19
Sum of permanent DMFT due to disease				5	4			16	5
Sum of permanent DMFT due to any cause				5	4			17	5
Bleeding_Percentage				10.43	14.37			7.88	11.26
Furcation_SUM				0.05	0.30			0.57	1.40
MAL				0.58	0.53			1.51	1.16

COPD: chronic obstructive pulmonary disease; DFMS: decayed, filled, missing surfaces; DFS: decayed, filled surfaces; DMFT: decayed, missing, filled teeth; MAL: mean attachment loss.

**Table 2:** Measuring the performance of machine learning and deep learning algorithms for predicting the COPD class (binary classification task). A k-fold cross-validation analysis was performed (k = 5). AUC: area under the curve; Accuracy: (TP + TN)/(TP + TN + FP + FN).

Algorithm	Accuracy	AUC
Logistic regression	0.884	0.835
Random forest classifier	0.883	0.819
SGD classifier	0.879	0.804
K-nearest neighbors	0.872	0.723
Decision tree classifier	0.823	0.608
GaussianNB	0.803	0.807
SVM	0.884	0.685
Custom CNN	0.887	0.953

## Data Availability

The dataset can be downloaded from the ‘NHANES’ database (https://wwwn.cdc.gov/nchs/nhanes/nhanes3/default.aspx). The python code and machine learning algorithm structures are available from: https://github.com/Freiburg-AI-Research.
